# Adoption of Preventive Measures During the Very Early Phase of the COVID-19 Outbreak in China: National Cross-sectional Survey Study

**DOI:** 10.2196/26840

**Published:** 2021-10-07

**Authors:** Joseph Lau, Yanqiu Yu, Meiqi Xin, Rui She, Sitong Luo, Lijuan Li, Suhua Wang, Le Ma, Fangbiao Tao, Jianxin Zhang, Junfeng Zhao, Dongsheng Hu, Liping Li, Guohua Zhang, Jing Gu, Danhua Lin, Hongmei Wang, Yong Cai, Zhaofen Wang, Hua You, Guoqing Hu, COVID-19 University Student Research Group

**Affiliations:** 1 Centre for Health Behaviours Research Jockey Club School of Public Health and Primary Care The Chinese University of Hong Kong Hong Kong Hong Kong; 2 Vanke School of Public Health Tsinghua University Beijing China; 3 School of Public Health Dali University Dali China; 4 Graduated School of Baotou Medical College Baotou Medical College Baotou China; 5 School of Public Health Xi’an Jiaotong University Health Science Center Xi'an China; 6 Department of Maternal, Child and Adolescent Health School of Public Health Anhui Medical University Hefei China; 7 School of Public Health Sichuan University Chengdu China; 8 Department of Psychology School of Education Henan University Kaifeng China; 9 Department of Biostatistics and Epidemiology School of Public Health Shenzhen University Health Science Center Shenzhen China; 10 Department of Epidemiology and Health Statistics College of Public Health Zhengzhou University Zhengzhou China; 11 Shantou University Medical College Shantou China; 12 Department of Psychology School of Psychiatry Wenzhou Medical University Wenzhou China; 13 Department of Medical Statistics School of Public Health Sun Yat-Sen University Guangzhou China; 14 Faculty of Psychology Beijing Normal University Beijing China; 15 School of Public Health Zhejiang University School of Medicine Hangzhou China; 16 School of Public Health Shanghai Jiao Tong University Shanghai China; 17 Public Health Department Qinghai University Medical College Xining China; 18 Department of Social Medicine and Health Education School of Public Health Nanjing Medical University Nanjing China; 19 Department of Epidemiology of Health Statistics Xiangya School of Public Health Central South University Changsha China; 20 See Authors’ Contributions

**Keywords:** COVID-19, health behavior, prevention, control, cognition, face mask, hand hygiene, interpersonal contacts, China, protection, public health, behavior, infectious disease, cross-sectional, survey

## Abstract

**Background:**

The outbreak of COVID-19 in China occurred around the Chinese New Year (January 25, 2020), and infections decreased continuously afterward. General adoption of preventive measures during the Chinese New Year period was crucial in driving the decline. It is imperative to investigate preventive behaviors among Chinese university students, who could have spread COVID-19 when travelling home during the Chinese New Year break.

**Objective:**

In this study, we investigated levels of COVID-19–related personal measures undertaken during the 7-day Chinese New Year holidays by university students in China, and associated COVID-19–related cognitive factors.

**Methods:**

A cross-sectional anonymous web-based survey was conducted during the period from February 1 to 10, 2020. Data from 23,863 students (from 26 universities, 16 cities, 13 provincial-level regions) about personal measures (frequent face-mask wearing, frequent handwashing, frequent home staying, and an indicator that combined the 3 behaviors) were analyzed (overall response rate 70%). Multilevel multiple logistic regression analysis was performed.

**Results:**

Only 28.0% of respondents (6684/23,863) had left home for >4 hours, and 49.3% (11,757/23,863) had never left home during the 7-day Chinese New Year period; 79.7% (19,026/23,863) always used face-masks in public areas. The frequency of handwashing with soap was relatively low (6424/23,863, 26.9% for >5 times/day); 72.4% (17,282/23,863) had frequently undertaken ≥2 of these 3 measures. COVID-19–related cognitive factors (perceptions on modes of transmission, permanent bodily damage, efficacy of personal or governmental preventive measures, nonavailability of vaccines and treatments) were significantly associated with preventive measures. Associations with frequent face-mask wearing were stronger than those with frequent home staying.

**Conclusions:**

University students had strong behavioral responses during the very early phase of the COVID-19 outbreak. Levels of personal prevention, especially frequent home staying and face-mask wearing, were high. Health promotion may modify cognitive factors. Some structural factors (eg, social distancing policy) might explain why the frequency of home staying was higher than that of handwashing. Other populations might have behaved similarly; however, such data were not available to us.

## Introduction

The World Health Organization (WHO) declared the COVID-19 outbreak a Public Health Emergency of International Concern on January 30, 2020 and declared a pandemic on March 11, 2020 [1,2]. Globally, there were 177.9 million infections and 3.8 million deaths (June 22, 2021), respectively [3]. In China, the outbreak coincided with the critical 7-day Chinese New Year holidays (January 25 to February 1), during which billions of trips were made across the country. On January 20, 2020, the government announced evidence of human-to-human transmission. Wuhan (the epicenter) was immediately locked down, and subsequently, other cities were also locked down [4]. Comprehensive control measures were enacted (eg, testing, quarantining, contact tracing, cancelling public events, closing of public areas, extending Chinese New Year holidays, and mandatory face-mask wearing [5,6]), and patients with COVID-19 were treated in more than 30 speedily built hospitals by medical specialists in different provinces across the country [7]. Many countries soon used similar standard strategies to combat COVID-19.

During the 7-day Chinese New Year period (January 25 to February 1), which began only 5 days after the start of the Wuhan lockdown (thus, during the very early phase of the outbreak among university students in China), the daily number of newly detected clinical or suspected COVID-19 infections in China reached its peak (n=17,959) on February 12, 2020, and then declined rapidly, to only 100 new infections on March 8 (ie, week 6 since the Wuhan lockdown) [8,9]. The daily figure for indigenous confirmed cases reached zero on March 22, 2020 [9] and remained very low afterward [10]. Thus, the first wave of the COVID-19 outbreak in mainland China had been effectively controlled within 6 to 8 weeks. The use of preventive behaviors might have led to the sharp decline. Worldwide, almost all governments have implemented some testing and social distancing measures [11]; their effectiveness, however, varied by country. Since March 2020, outbreaks have occurred in every continent [3,12].

Effective control of severe acute respiratory syndrome (SARS) outbreaks depended on community-level behavioral responses (ie, general adoption of protective measures, such as face-mask use, good hand hygiene, and social distancing [13,14]). Unfortunately, strong public health messages (eg, the importance of urgent uptake of the aforementioned preventive measures) throughout the COVID-19 pandemic have been blurred by politics and have not always been well received by governments and citizens in many countries. The identification of factors associated with preventive behaviors facilitates health promotion to improve preventive behaviors. Cognitive factors related to SARS and influenza A (H1N1) of preventive measures during such epidemic periods have been identified (eg, perceived risk of SARS infection) [13-19]. Similar factors have been identified during the COVID-19 pandemic [20-22].

The rationale of this study was to understand and document the behavioral responses among university students during the very early phase of the COVID-19 outbreak in China. During the Chinese New Year (the peak travel season in China), university students could have transmitted and spread the virus across the country because of their travel during that time period. It is essential to examine Chinese university students’ behavioral responses to understand why their travel movements did not appear to cause COVID-19 outbreaks countrywide. There is a dearth of such studies. Although generalization to the general population is impossible, the findings are potentially illustrative of what might have occurred in other populations in China, and thus, might to some extent provide some clues about how the pandemic was controlled within a short period of time in China.

We aimed to investigate the frequency with which 3 key preventive measures—face-mask wearing, frequent handwashing, and frequent home staying—were practiced, as well as related cognitive factors among university students across mainland China during the critical 7-day Chinese New Year period.

## Methods

### Study Design

A cross-sectional anonymous web-based survey was conducted during the period from February 1 to 10, 2020. At the time of the study, there were a total of 1272 universities in China. Convenience sampling was used to select the same faculties (medicine, arts, science, engineering, social science, and others) from 26 universities (16 cities in 13 out of 32 provinces, municipalities, or autonomous regions); altogether, selected faculties contained 36,560 students, who were invited through WeChat to complete an anonymous web-based questionnaire, which included an informed consent statement (invitations per university: median 1165, IQR 2271). Methodological details have been previously described in other papers based on the same data set [23,24].

A total of 25,647 participants returned completed questionnaires. The overall response rate was 70.2% (25,647/36,560). The mean response rate for cities was 70% (SD 17%), with 12 of the 16 cities having response rates >60%, while the average response rate for the 26 universities was 68.4%. Of 25,647 completed questionnaires, 1784 were excluded (due to failure to pass the built-in consistency checks: n=1197; respondents were diagnosed with COVID-19: n=47; respondents were quarantined due to COVID-19 exposure: n=515; respondents had been outside mainland China during the 7-day Chinese New Year period: n=25); therefore, the effective sample size for analysis was 23,863 (93.0%). Ethics approval was obtained from the Chinese University of Hong Kong.

### Measures

#### Background Information

Background information about sociodemographic characteristics, school, where respondents were located at the time of the survey (ie, staying in their universities’ cities or with the family), self-perceived physical health status, living arrangements during the 7-day Chinese New Year holidays (ie, whether staying in their universities’ cities and whether staying with their family from January 25 to February 1, 2020), and the lockdown status of the community where they were located at the time of the survey.

#### Dependent Variables

We assessed the frequency of uptake of various preventive measures during the period from January 25 to February 1, 2020 (ie, the 7-day Chinese New Year holidays). Frequency of face-mask wearing was assessed with the question: “Have you worn a mask when going out, no matter whether you have symptoms or not?” Respondents answered using a scale from 1 (definitely no) to 5 (definitely yes), which was recoded into 1 (definitely yes) and 0 (other responses). Daily frequency of handwashing with water, soap, or disinfectant was assessed, with 0 to 2, 3 to 5, 6 to 10, 11 to 15, >15 times as response options; responses were then categorized as frequent (>10) or less frequent (0-10). Frequency of home staying was assessed, with 0 to 4 hours or >4 hours at home during the 7-day of the Chinese New Year as response options; responses were recoded into 1 (frequent) and 0 (less frequent). In addition, we used a composite Preventive Measure Indicator—the number of abovementioned measures that were frequently used—which was recoded into a binary variable: low (0 or 1 frequently used preventive measures) or high (2 or 3 frequently used preventive measures).

#### Independent Variables

We assessed a set of cognitive variables: Perceived Probable Transmission Mode Indicator (the number of appropriate answers about transmission by droplets, touching infected persons, and touching contaminated objects), which ranged from 0 to 3; perceived asymptomatic transmission knowledge (yes or no/don’t know); perceived severity of COVID-19 (ie, “Whether COVID-19 would easily cause permanent bodily damage?”), with responses 0 (disagree/don’t know) or 1 (agree); perceived risk of contracting COVID-19 in the upcoming year for oneself, family members, and peers, with responses 1 (extremely high/high) or 0 (other); Perceived Risk Indicator (the number of responses equal to 1), which ranged from 0 to 3; perceived nonavailability of effective vaccines, with responses 1 (agree) or 0 (else); and perceived nonavailability of effective vaccine-specific treatment for COVID-19, with responses 1 (agree) or 0 (else).

An Efficacy of Personal Preventive Measure Indicator was obtained by summing 5 scores (frequent face-mask wearing in public areas, frequent handwashing with water, frequent handwashing with soap or disinfectant, household sterilization, and avoiding going to crowded places), where scores less than 15 (0th to 26.4th percentile) were low, scores from 16 to 18 (26.4th to 58.4th percentile) were medium, and scores equal to 19 or 20 (58.4th to 100th percentile) were high. The Efficacy of Governmental Preventive Measure Indicator was obtained by summing 6 measures (cancellation of public events, lockdown of Wuhan, closing public venues such as restaurants and cinemas, home staying, primary school to university class suspension, and mandatory face-mask wearing in public areas), where scores less than 20 (27.4th percentile) were low, scores from 21 to 23 (27.4th -51.4th percentile) were medium, and a score of 24 (51.4th to100th percentile) was high.

### Statistical Analysis

Pooled proportions were estimated using meta-analysis techniques that consider random-effects and inverse variance weighting (universities were the pooling units) [25]. Simple (univariate) logistic regression was performed to examine the crude association between background variables and dependent variables. Significant background variables (*P*<.05) were potential confounders of associations between cognitive variables and dependent variables and were adjusted for in subsequent multivariable logistic regression analysis. Multilevel logistic regression models with random effects, adjusted for background variables, were fit separately for the 4 preventive measure–dependent variables (with university as the first level). Summary models included all independent variables. Adjusted odds ratios (OR) with 95% confidence intervals are reported. SPSS statistical software (version 25; IBM Corp) and R software (version 3.5.2; the R Project) were used. Significance was defined as a *P* value <.05.

## Results

### Background Characteristics

At the time of the survey, 53.4% of the participants (12,747/23,863) were located in the same city as their university, 93.5% were staying with their family (22,304/23,863), 70.6% were in communities under lockdown, and 79.4% (18,937/23,863) self-reported good or very good physical health status (Table 1).

**Table 1:** Background variable descriptive statistics.

Variables			Respondents (n=23,863), n (crude %)	Pooled % (95% CI)
**Sociodemographics**				
	**Gender**			
		Male	7605 (31.9)	29.8 (26.2, 33.4)
		Female	16,258 (68.1)	70.2 (66.6, 73.8)
	**Grade**			
		First year	9017 (37.8)	36.8 (23.7, 49.9)
		Second year	6425 (26.9)	27.5 (21.6, 33.3)
		Third year	5061 (21.2)	21.0 (13.7, 28.3)
		Fourth year	2281 (9.6)	7.3 (6.2, 8.4)
		Fifth year	542 (2.3)	0.8 (0.5, 1.1)
		Master or above	537 (2.3)	1.0 (0.6, 1.3)
	**Major**			
		Medicine	10,850 (45.5)	34.5 (27.9, 41.2)
		Arts	4232 (17.7)	22.3 (18.1, 26.4)
		Science	3901 (16.4)	15.1 (11.6, 18.6)
		Engineering	1809 (7.6)	8.8 (7.0, 10.5)
		Social science	846 (3.6)	4.1 (3.2, 5.0)
		Other	2225 (9.3)	8.5 (6.6, 10.5)
**Living arrangement during Chinese New Year**				
	**Staying in the same city as their university**			
		No	11,116 (46.6)	48.1 (42.3, 53.8)
		Yes	12,747 (53.4)	51.9 (46.2, 57.7)
	**Staying with family**			
		No	1559 (6.5)	5.6 (4.7, 6.5)
		Yes	22,304 (93.5)	94.4 (93.5, 95.3)
**Self-reported physical health status**				
	Moderate/poor/very poor		4926 (20.6)	22.5 (20.4, 24.7)
	Good/very good		18,937 (79.4)	77.5 (75.3, 79.6)
**Local entry and exit control (ie, lockdown)**				
	No		7081 (29.4)	34.1 (27.2, 40.9)
	Yes		16,845 (70.6)	65.9 (59.1, 72.8)

### Preventive Measures During the Chinese New Year Period

The majority of respondents (19,026/23,863, 79.7%) always used face masks when going out without flu symptoms (frequent face-mask users); only 7.7% (1842/23,863) had never used face masks during the Chinese New Year (744/1842, 40.4% of whom had not left home). Approximately three-quarters (72.0%) went out for <4 hours per day (0 hours: 11,757/23,863, 49.3%; 1-4 hours: 5422/23,863, 22.7%) during the 7-day period. Approximately three-quarters (17,439/23,863, 73.1%) washed their hands with soap or disinfectant for 0 to 5 times per day, 28.6% (6836/23,863) washed their hands with soap or disinfectant 0 to 2 times per day, and 43.7% (10,440/23,863) washed hands with either water or soap or disinfectant >10 times per day. Of the 23,863 respondents, 44.7% (10,675/23,863) and 27.7% (6607/23,863) had frequently taken up 2 and 3 key preventive measures, respectively (Table 2).

**Table 2:** Descriptive statistics for behavioral variables related to COVID-19 among university students in China.

Variables		Respondents (n=23,863), n (crude %)	Pooled % (95% CI)
**Frequent face-mask wearing when went out**			
	Not definitely yes	4837 (20.3)	16.7 (13.7, 19.8)
	Definitely yes	19,026 (79.7)	83.3 (80.2, 86.3)
**Frequent handwashing (frequencies of washing either soap or water)**			
	0-10 times/day	13,423 (56.3)	55.6 (52.9, 58.2)
	>10 times/day	10,440 (43.7)	44.4 (41.8, 47.1)
**Frequent home staying (total number of hours went out during the 7-day Chinese New Year period)**			
	>4 hours	6684 (28.0)	27.5 (25.1, 29.8)
	1-4 hours	5422 (22.7)	25.0 (23.4, 26.7)
	Never went out	11,757 (49.3)	46.9 (43.6, 50.2)
**Preventive Measure Indicator^a^**			
	0-1	6581 (27.6)	25.2 (21.8, 28.5)
	2-3	17,282 (72.4)	74.8 (71.5, 78.2)

^a^The Preventive Measure Indicator counted the number of the frequently used preventive measures (ie, frequent face-mask wearing, frequent handwashing, and frequent home staying.

### COVID-19–Related Cognitions

A majority (21,991/23,863, 92.2%) indicated ≥2 of the 3 key modes of transmission (droplets, touching infected persons, and touching contaminated surfaces) and perceived possibility of asymptomatic transmission (19,549/23,863, 81.9%). Less than 20% (3238/23,863, 13.6%) believed that there was a high risk of themselves, their family members, or their peers contracting the virus; 35.7% (8523/23,863) perceived that COVID-19 would easily cause permanent bodily damage. Approximately 70.0% (18,281/23,863 and 15,015/23,863, respectively) perceived effective vaccines and specific treatments for COVID-19 were not available, and personal and governmental preventive measures were perceived to be highly effective (Table 3).

**Table 3:** Descriptive statistics for cognitive variables related to COVID-19 among university students in China.

Variables				Respondents (n=23,863), n (crude %)		Pooled % (95% CI)
**Transmission-related variables**						
	**Perceived Probable Transmission Mode Indicator (number of appropriate answers)**					
		0	292 (1.2)		0.9 (0.6, 1.2)	
		1	1580 (6.6)		6.2 (5.2, 7.1)	
		2	6171 (25.9)		26.3 (24.1, 28.6)	
		3	15,820 (66.3)		66.3 (63.5, 69.1)	
	**Perceived asymptomatic transmission**					
		No or don’t know	4314 (18.1)		16.6 (13.7, 19.6)	
		Yes	19,549 (81.9)		83.4 (80.4, 86.3)	
**Perceived severity**						
	**Permanent bodily damage**					
		Disagree/don’t know	15,340 (64.3)		66.4 (64.6, 68.2)	
		Agree	8523 (35.7)		33.6 (31.8, 35.4)	
**Perceived risk**						
	**Perceived Risk Indicator**					
		0	18,779 (78.7)		77.4 (75.2, 79.6)	
		1	1846 (7.7)		8.0 (7.0, 8.9)	
		2	1707 (7.2)		7.2 (6.2, 8.2)	
		3	1531 (6.4)		6.6 (5.8, 7.4)	
**Medical preparedness**						
	**Perceived nonavailability of vaccines**					
		Disagree or don’t know	5582 (23.4)		19.9 (16.4, 23.4)	
		Agree	18,281 (76.6)		80.1 (76.6, 83.6)	
	**Perceived nonavailability of specific treatment**					
		Disagree or don’t know	8848 (37.1)		34.5 (31.3, 37.8)	
		Agree	15,015 (62.9)		65.5 (62.2, 68.7)	
**Perceived efficacy**						
	**Efficacy of Personal Preventive Measure Indicator**					
		≤15 (<26.4th percentile)	6298 (26.4)		24.4 (22.4, 26.4)	
		16-18 (26.4th to 58.4th percentile)	7646 (32.0)		33.4 (31.5, 35.2)	
		19-20 (>58.4th percentile)	9919 (41.6)		41.8 (39.0, 44.5)	
	**Efficacy of Governmental Preventive Measure Indicator**					
		≤ 20 (<27.4th percentile)	6528 (27.4)		25.4 (23.1, 27.7)	
		21-23 (27.4th to 51.4th percentile)	5729 (24.0)		25.2 (22.9, 27.4)	
		24 (>51.4th percentile)	11,606 (48.6)		49.2 (45.3, 53.1)	

### Background Factors

Female university students were more likely than male university students to have frequently used more preventive measures (univariate OR 1.40). University year, major, staying with family, staying in the same city as their university, self-reported physical health, and lockdown were associated with some or all of the 4 dependent variables (Table 4); subsequent analyses were adjusted for these background variables as they were potential confounders of the associations between the cognitive factors and preventive behaviors.

**Table 4:** Univariate associations (crude odds ratios) between the background variables (sociodemographics, living arrangement during the Chinese New Year, self-perceived health, and local lockdowns) and preventive measures among university students in China (n=23,863).

Independent variables					Dependent variable, univariate odds ratio (95% CI)						
			Frequent face-mask wearing			Frequent handwashing		Frequent home staying		Preventive measure indicator	
**Sociodemographic**											
	**Gender**										
		Male	1.00			1.00		1.00		1.00	
		Female	1.68 (1.48-1.91)***			1.15 (1.06-1.26)**		1.10 (1.04-1.15)***		1.40 (1.31-1.51)***	
	**Grade**										
		First year	1.00			1.00		1.00		1.00	
		Second year	1.12 (1.00-1.25)			1.07 (0.99-1.15)		0.95 (0.88-1.04)		1.05 (0.97-1.15)	
		Third year	1.15 (1.04-1.28)**			1.13 (1.01-1.26)*		0.97 (0.90-1.05)		1.12 (1.00-1.25)	
		Fourth year	1.15 (1.01-1.30)*			1.11 (0.97-1.26)		0.94 (0.86-1.04)		1.01 (0.01-1.11)	
		Fifth year	1.33 (0.96-1.85)			1.19 (1.05-1.34)**		0.77 (0.66-0.91)**		1.10 (0.80-1.51)	
		Master or above	1.45 (1.03-2.06)*			1.35 (1.26-1.45)***		0.79 (0.71-0.88)***		1.19 (0.96-1.47)	
	**Major**										
		Medicine	1.00			1.00		1.00		1.00	
		Arts	1.00 (0.89-1.13)			1.00 (0.93-1.06)		1.04 (0.94-1.14)		1.04 (0.97-1.12)	
		Science	0.73 (0.66-0.81)***			0.96 (0.90-1.02)		0.91 (0.85-0.98)*		0.82 (0.75-0.90)***	
		Engineering	0.76 (0.69-0.84)***			0.85 (0.77-0.94)**		0.93 (0.83-1.05)		0.80 (0.70-0.92)**	
		Social science	0.88 (0.73-1.05)			0.84 (0.75-0.94)**		0.82 (0.69-0.98)*		0.77 (0.63-0.94)**	
		Other	0.99 (0.89-1.10)			0.99 (0.92-1.07)		1.00 (0.88-1.14)		0.97 (0.89-1.06)	
**Living arrangement during the Chinese New Year**											
	**Staying in the same city as their university**										
		No	1.00			1.00		1.00		1.00	
		Yes	0.93 (0.87-0.98)*			0.93 (0.87-0.99)*		0.77 (0.73-0.83)***		0.82 (0.76-0.87)***	
	**Staying with the family**										
		No	1.00			1.00		1.00		1.00	
		Yes	1.26 (1.10-1.45)**			0.72 (0.65-0.81)***		0.77 (0.65-0.92)**		0.91 (0.77-1.07)	
**Self-perceived physical health status**											
	Moderate/poor/very poor			1.00			1.00		1.00		1.00
	Good/very good			1.42 (1.33-1.51)***			1.34 (1.27-1.42)***		1.30 (1.23-1.38)***		1.48 (1.36-1.61)***
**Local lockdown (entry/exit control)**											
	No			1.00			1.00		1.00		1.00
	Yes			0.93 (0.81-1.06)			1.00 (0.92-1.09)		1.21 (1.14-1.28)***		1.06 (0.97-1.15)

*P<.05.

**P<.01.

***P<.001.

### Adjusted Associations Between Cognitive Factors and Preventive Measures

Perceived knowledge about probable modes of transmission of COVID-19 was significantly associated with the number of frequently used preventive measures (adjusted OR ranged from 2.50 to 3.06) and frequent face-mask wearing (adjusted OR ranged from 4.32 to 6.25) (Table 5). Perceived knowledge about asymptomatic transmission was associated with frequent face-mask wearing (adjusted OR 1.54, 95% CI 1.34-1.76) and more frequently used preventive measures (adjusted OR 1.27, 95% CI 1.18-1.36). Perceived permanent bodily damage was mildly associated with frequent face-mask wearing (adjusted OR 1.24, 95% CI 1.14-1.35), frequent handwashing (adjusted OR 1.07, 95% CI 1.02-1.12), and the number of frequently used preventive measures (adjusted OR 1.17, 95% CI 1.11-1.22), but was not associated with frequent home staying (adjusted OR 1.02, 95% CI 0.96-1.08). Perceived risks of infection was associated with less preventive behaviors (adjusted OR ranged from 0.76 to 0.83).

Perceived nonavailability of vaccines or specific treatments was significantly and mildly associated with frequent face-mask wearing (adjusted OR 1.43 and 1.27, respectively) and number of frequently used preventive measures (adjusted OR 1.18 and 1.15, respectively). Perceived efficacy of the personal measures was strongly associated with frequent face-mask wearing (adjusted OR ranged from 1.86 to 3.51) and number of frequently used preventive measures (adjusted OR ranged from 1.44 to 2.08), and mildly with frequent handwashing (adjusted OR ranged from 1.07 to 1.33) and frequent home staying (adjusted OR ranged from 1.05 to 1.14). Perceived efficacy of governmental measures was similarly associated with the 4 dependent variables.

A summary logistic regression model, which contained all the independent variables and was adjusted for background variables (potential confounders), exhibited largely similar results (Multimedia Appendix 1).

**Table 5:** Adjusted associations between cognitive factors and preventive measures among university students in China (n=23,863).

Independent variables				Dependent variables, adjusted odds ratio^a^ (95% CI)			
			Frequent face-mask wearing		Frequent handwashing	Frequent home staying	Preventive measure indicator
**Transmission-related variables**							
	**Perceived Probable Transmission Mode Indicator (No. of appropriate answers)**						
		0	1.00		1.00	1.00	1.00
		1	4.32 (2.81-6.65)***		0.89 (0.68-1.17)	1.25 (0.92-1.69)	2.50 (2.00-3.12)***
		2	5.31 (3.31-8.54)***		0.89 (0.74-1.07)	1.27 (0.91-1.79)	2.62 (2.03-3.38)***
		3	6.25 (3.77-10.36)***		1.01 (0.84-1.21)	1.33 (0.96-1.83)	3.06 (2.35-3.98)***
	**Perceived asymptomatic transmission**						
		No/don’t know	1.00		1.00	1.00	1.00
		Yes	1.54 (1.34-1.76)***		1.03 (0.98-1.09)	1.08 (1.00-1.18)	1.27 (1.18-1.36)***
**Perceived severity**							
	**Permanent bodily damage**						
		Disagree/don’t know	1.00		1.00	1.00	1.00
		Agree	1.24 (1.14-1.35)***		1.07 (1.02-1.12)*	1.02 (0.96-1.08)	1.17 (1.12-1.22)***
**Perceived risk**							
	**Perceived Risk Indicator**						
		0	1.00		1.00	1.00	1.00
		1	0.81 (0.74-0.90)***		0.95 (0.91-1.00)*	0.84 (0.75-0.95)**	0.83 (0.73-0.94)**
		2	0.78 (0.71-0.85)***		0.90 (0.78-1.03)	0.80 (0.71-0.92)**	0.76 (0.68-0.85)***
		3	0.82 (0.63-1.06)		0.94 (0.86-1.03)	0.76 (0.67-0.87)***	0.76 (0.67-0.86)***
**Medical preparedness**							
	**Perceived nonavailability of vaccines**						
		Disagree/don’t know	1.00		1.00	1.00	1.00
		Agree	1.43 (1.32-1.54)***		1.05 (1.00-1.10)	1.00 (0.92-1.07)	1.18 (1.10-1.28)***
	**Perceived nonavailability of specific treatment**						
		Disagree/don’t know	1.00		1.00	1.00	1.00
		Agree	1.27 (1.18-1.36)***		1.00 (0.94-1.05)	1.02 (0.97-1.08)	1.15 (1.09-1.21)***
**Perceived efficacy of preventive measures**							
	**Efficacy of Personal Preventive Measure Indicator**						
		≤15 (26.4 percentile)	1.00		1.00	1.00	1.00
		16-18 (58.4 percentile)	1.86 (1.62-2.14)***		1.07 (1.01-1.14)*	1.05 (0.99-1.12)	1.44 (1.33-1.55)***
		19-20 (100 percentile)	3.51 (3.06-4.02)***		1.33 (1.25-1.43)***	1.14 (1.08-1.21)***	2.08 (1.93-2.23)***
	**Efficacy of Governmental Preventive Measure Indicator**						
		≤20 (27.4 percentile)	1.00		1.00	1.00	1.00
		21-23 (51.4 percentile)	2.07 (1.82-2.36)***		1.10 (1.03-1.18)**	1.04 (0.97-1.12)	1.15 (1.35-1.65)***
		24 (100 percentile)	4.05 (3.58-4.58)***		1.28 (1.22-1.35)***	1.17 (1.11-1.22)***	2.23 (2.10-2.36)***

^a^Adjusted for gender, grade, major, living arrangement during Chinese New Year, self-perceived physical health status, and local entry and exit control.

*P<.05.

**P<.01.

***P<.001.

## Discussion

We found that some preventive behaviors, especially social distancing (staying at home) and face-mask use, were frequently practiced by the majority of university student respondents during the 7-day Chinese New Year holiday week. Cognitive factors—perceived knowledge about probable modes of transmission and perceived knowledge about asymptomatic transmission, perceived permanent bodily damage, perceived nonavailability of vaccines or specific treatment, perceived efficacy of the personal measures, and perceived efficacy of governmental measures—were associated with practicing the 3 preventive behaviors. Unexpectedly, perceived risks of infection was associated with less preventive behaviors.

One of the key findings was the amount that people remained home during the 7-day Chinese New Year holiday period. The time frame for investigating preventive behaviors in the very early phase of the COVID-19 outbreak in China was set during the holiday period (which was 2-9 days into the Wuhan lockdown). This holiday period is typically filled with travel, celebrations, dining, gatherings, open markets, and mutual family visits, which would have entailed a very high risk of COVID-19 transmission and spread across provinces via university students when they traveled home. Such risks, however, seem to have been mitigated—the majority of university students stayed home all or most of the time and frequently wore face masks in public areas. As the responses from quarantined individuals were excluded from the study and there was, then, no penalty for going out, most home staying was likely to be voluntary, although possibly based on governmental advice. Frequent home staying was not used to control SARS and H1N1; the massive scale of voluntary home staying is unprecedented. Consistent face-mask wearing was much higher than the 61.2% to 64.3% recorded during the SARS period in Hong Kong [26,27]. Neither social norms nor governmental policy about the use of face masks existed during the initial period of the COVID-19 outbreak in China, which suggests that, to some extent, spontaneous behavioral responses to practice COVID-19 preventive measures might have commonly occurred among university students nationwide, as 31 of the 32 provincial-level regions in China were represented by respondents. The potential spontaneity is remarkable as only a low number of 381 newly confirmed infections were detected outside Hubei province (where Wuhan is located) on February 10 [28]. It is notable that good knowledge about the key transmission modes and high perceived efficacies of personal and governmental measures in preventing COVID-19 were significantly associated with the number of frequently used preventive measures (all *P*<.001), indicating that it is potentially important to disseminate information to increase COVID-19–related knowledge to promote positive behavioral responses.

The findings further suggest that health education about transmission via fomites is required, as the level of washing hands with soap or disinfectants was much lower than that of mask-wearing. The frequency of handwashing could be improved, as 73.1% (17,439/23,863) had only washed their hands with soap or disinfectant for 0 to 5 times per day, compared to 91.4% of the Hong Kong general public had washed hands for >6 times per day to prevent H1N1 [15]. Handwashing was widely publicized during the SARS period in Hong Kong [29]. Interestingly, handwashing was less practiced than home staying and face-mask wearing, possibly because handwashing is a privately performed behavior while home staying and face-mask wearing are visible behaviors that protect both the doer and other people. Handwashing might be less subjected to social norms and controls.

Corroborating literature with respect to knowledge about COVID-19, perceived permanent bodily damages (severity) [30], perceived nonavailability of vaccines or specific treatments [31], perceived efficacy of personal measures, and perceived efficacy of governmental measures [32] were associated with practicing preventive measures frequently. Unexpectedly, high perceived risk of infection was mildly associated with fewer preventive behaviors. As the use of preventive measures may reduce perceived risk, cross-sectional studies have often reported similar negative associations (eg, association between condom use and lower perceived risk of contracting HIV [33]). It is interesting that cognitive factors exhibited stronger associations with frequent face-mask wearing than frequent home staying. It is plausible that because some governmental social distancing measures (eg, lockdown, extended holidays, suspending events, and closing venues) had removed reasons to go out and the government encouraged staying at home (eg, for personal safety, to be a good citizen, and to contribute to controlling the national pandemic), home staying may hence be influenced less by individual-level cognitive factors than face-mask wearing and more so by structural policy factors and interpersonal norm factors. Such a contention needs to be confirmed in the future. If true, global public health workers may need to pay more attention to the structural and interpersonal factors in controlling the COVID-19 pandemic.

It is noteworthy that 70% of the respondents (16,845/23,863) reported some entry and exit restrictions in their communities. It is possible that social distancing policies had already been implemented in many parts of China very soon after the COVID-19 outbreak. Other studies [34-36] have also reported high levels of preventive behaviors in different populations in China (eg, general population, factory workers, and teachers) from February to May 2020. We speculate that the nation started responding to COVID-19 shortly after the initial outbreak. This study has thus documented active positive behavioral responses to COVID-19 in one important population of university students in China, while social distancing and face-mask use remain controversial even now, due to potential infringement on personal freedoms, in many countries [37-40].

The study has some limitations. Although it included respondents from universities in 16 cities in 13 provinces, this still did represent truly national coverage. Selection bias may exist, as classes and departments were not randomly selected. We were unable to include universities in Hubei Province, the epicenter. The sample only included university students and cannot be generalized to other populations. The subsample sizes of the participating universities varied; the weighted data were very similar to the observed frequencies. The uptake of preventive measures was self-reported and refined measurement was not allowed (eg, the exact amount of time spent outside the home). We did not cover important interpersonal factors (eg, subjective norms and social support), which are associated with many health-related behaviors [41]. The cross-sectional study design does not allow for causal inferences. In addition, information bias might exist due to recall and social desirability.

The sample of university students in China demonstrated very strong behavioral prevention responses, especially home staying (social distancing) and face mask use, during the initial phase of the COVID-19 outbreak in China. Such preventive behaviors may have averted subsequent outbreaks that could have arisen from a large volume of nationwide travel by university students during Chinese New Year holidays. Potential determinants of the preventive behaviors were identified. Prompt health education, given the findings of this study, should be provided to university students through social media in the very early phase of outbreaks of future pandemics. The strong behavioral responses that were observed might be, to some extent, spontaneous and may indicate the importance of structural factors (eg, strong governmental policies, mobilization, and social capital). Although there were substantial hardships during the early phase of the pandemic, the entire country remained supportive, united, orderly, and harmonious—altruism and patriotism appeared to be wide-spread [42]. Good social capital (eg, trust in the government and mutual help) might have played an important role in initiating the positive responses among university students. Further studies are warranted to understand the roles of social capital in controlling the spread of COVID-19 infections in and outside China. 

## Appendix

Multimedia Appendix 1 Summary models entering all cognitive factors as independent variables (n=23,863).

## Abbreviations

H1N1: influenza A
HIV: human immunodeficiency virus
OR: odds ratio
SARS: severe acute respiratory syndrome
WHO: World Health Organization
