# Supplementary material for: Adoption of Preventive Measures During the Very Early Phase of the COVID-19 Outbreak in China: National Cross-sectional Survey Study
Source: JMIR Public Health Surveill. 2021 Oct 7;7(10):e26840. doi: 10.2196/26840 (PMC8500352; doi:10.2196/26840)
Supplement: Multimedia Appendix 1 [file publichealth_v7i10e26840_app1.docx]

Multimedia appendix 1. Summary models entering all cognitive factors as independent variables (N=23,863)

|  | Dependent variables | | | |
| --- | --- | --- | --- | --- |
| Independent variables | Frequent face-mask wearing | Frequent  handwashing | Frequent  home-staying | Preventive Measure Indicator |
|  | ORm (95% CI) | ORm (95% CI) | ORm (95% CI) | ORm (95% CI) |
| **Transmission-related variables** |  |  |  |  |
| Perceived Probable Transmission Mode Indicator (No. of appropriate answers) |  |  |  |  |
| 0 | 1.00 | 1.00 | 1.00 | 1.00 |
| 1 | 2.71(1.81-4.06)*** | 0.82(0.62-1.08) | 1.15(0.83-1.57) | 1.84(1.49-2.28)*** |
| 2 | 3.04(1.94-4.78)*** | 0.81(0.67-0.97)* | 1.16(0.80-1.68) | 1.83(1.43-2.35)*** |
| 3 | 3.38(2.08-5.49)*** | 0.90(0.75-1.08) | 1.21(0.84-1.73) | 2.07(1.59-2.70)*** |
| Perceived asymptomatic transmission |  |  |  |  |
| No/don’t know | 1.00 | 1.00 | 1.00 | 1.00 |
| Yes | 1.21(1.13-1.30)*** | 1.01(0.96-1.06) | 1.06(0.96-1.17) | 1.11(1.04-1.18)** |
| **Perceived severity** |  |  |  |  |
| Permanent bodily damage |  |  |  |  |
| Disagree/don’t know | 1.00 | 1.00 | 1.00 | 1.00 |
| Agree | 1.09(0.97-1.21) | 1.04(0.99-1.10) | 1.01(0.96-1.07) | 1.09(1.03-1.15)** |
| **Perceived risk** |  |  |  |  |
| Perceived Risk Indicator |  |  |  |  |
| 0 | 1.00 | 1.00 | 1.00 | 1.00 |
| 1 | 0.80(0.73-0.87)*** | 0.96(0.91-1.00) | 0.84(0.75-0.94)** | 0.82(0.73-0.92)** |
| 2 | 0.74(0.68-0.82)*** | 0.89(0.78-1.03) | 0.80(0.70-0.91)** | 0.75(0.67-0.83)*** |
| 3 | 0.82(0.68-1.00) | 0.93(0.85-1.03) | 0.76(0.67-0.87)*** | 0.75(0.68-0.83)*** |
| **Medical preparedness** |  |  |  |  |
| Perceived non-availability of vaccines |  |  |  |  |
| Disagree/don’t know | 1.00 | 1.00 | 1.00 | 1.00 |
| Agree | 1.17(1.10-1.25)*** | 1.04(1.00-1.09) | 0.97(0.89-1.05) | 1.05(0.97-1.14) |
| Perceived non-availability of specific treatment |  |  |  |  |
| Disagree/don’t know | 1.00 | 1.00 | 1.00 | 1.00 |
| Agree | 1.08(0.98-1.25) | 0.97(0.92-1.02) | 1.02(0.97-1.07) | 1.05(1.00-1.12) |
| **Perceived efficacy of preventive measures** |  |  |  |  |
| Efficacy of Personal Preventive Measure Indicator |  |  |  |  |
| <=15 (26.4 percentile) | 1.00 | 1.00 | 1.00 | 1.00 |
| 16-18 (58.4 percentile) | 1.14(1.04-1.25)*** | 1.02(0.94-1.11) | 0.99(0.93-1.06) | 1.08(1.03-1.13)** |
| 19-20 (100 percentile) | 1.61(1.48-1.77)*** | 1.22(1.10-1.36)*** | 1.04(0.96-1.12) | 1.33(1.21-1.48)*** |
| Efficacy of Governmental Preventive Measure Indicator |  |  |  |  |
| <=20 (27.4 percentile) | 1.00 | 1.00 | 1.00 | 1.00 |
| 21-23 (51.4 percentile) | 1.69(1.51-1.88)*** | 1.04(0.96-1.13) | 1.03(0.95-1.11) | 1.32(1.19-1.45)*** |
| 24 (100 percentile) | 2.85(2.55-3.18)*** | 1.13(1.03-1.23)** | 1.13(1.05-1.21)** | 1.79(1.65-1.94)*** |

Notes: Explanations of variable definitions are given in footnotes of Table 1; ORm were derived from multi-level multiple logistic regression analyses entering all cognitive factors as independent variables along with adjusted background variables (sex, grade, faculty, living arrangement during CNY, self-perceived physical health status, and local entry/exit control); *, p<.05; **, p<.01; ***, p<.001. Abbreviation: ; ORm (multivariable odds ratio); CI (confidence interval); CNY (Chinese New Year)
